# Impacts of an intensive community‐based support for patients with severe or morbid obesity in the United Kingdom: A qualitative study

**DOI:** 10.1002/puh2.44

**Published:** 2022-12-25

**Authors:** Mick Bond, Annette Haywood, Eleanor Holding, Elizabeth Goyder, Paul Bissell, John Stephenson, Rachel Holt

**Affiliations:** ^1^ School of Health and Related Research (ScHARR), University of Sheffield Sheffield UK; ^2^ School of Human and Health Sciences, University of Huddersfield Huddersfield UK

**Keywords:** community‐based team, obesity, weight management

## Abstract

**Background**: To explore the long‐term impact of a UK specialist service providing intensive weight management support for patients with severe and complex obesity. An interview‐based study of former patients about their contact with the service and how it had affected their subsequent weight management. The questions covered areas of difference between those who had received lifestyle change support and those who had also had weight loss surgery.

**Methods**: This qualitative study took place with former patients of a community‐based weight management service for people with severe or morbid obesity who had been discharged for at least 2 years. Nineteen interviews took place with patients initially contacted by questionnaire about their experiences of involvement with the service. Participants interviewed all had support to change their lifestyle, and some also had bariatric surgery. A narrative interview approach was employed to explore participants’ weight management after involvement with the service.

**Results**: Most participants maintained their weight loss. The self‐esteem of some participants had significantly improved. For some, the weight loss came with significant problems in terms of dietary restrictions and loose skin. Participants said they would like more open‐ended contact, particularly for those who had bariatric surgery.

**Conclusions**: The study supported current research findings that both those who had bariatric surgery or who just had lifestyle change support experienced a significant reduction in their weight. In this respect, participants thought it was a success. However, for a number, it was at a price in terms of the amount and type of food they could eat. The study adds to the understanding of the impact of bariatric surgery and weight management support from the patients’ perspective and of the support needs of patients having lifestyle support and surgery to help them manage the long‐term impact of obesity and treatment side‐effects.

## INTRODUCTION

The UK ranks 10th in the Organisation for Economic Co‐operation and Development (OECD) prevalence figures for obesity. In 2020 26% of men and 29% of women were obese in the UK [1]. In 2018/19, there were 11,117 hospital admissions with a primary diagnosis of obesity. Obesity was recorded as a co‐morbid condition in a further 876,000 admissions of obesity. At a local level, in the English county with a population of 807,183, where the study took place, 63.8% of adults were overweight or obese, which is significantly more than the figure of 61.3% for England as a whole [2].

Obesity has a considerable health and economic impact on the National Health Service (NHS), with official estimated yearly costs of £5 billion in the same year. It significantly increases the risk of developing co‐morbidities such as type 2 diabetes, coronary artery disease and cancer, and may reduce life expectancy by 11 years [4]. Additionally, people can experience obesity‐related psychosocial consequences, with discrimination, anxiety and depression having a further impact on individuals and society [4]. Literature on the effect of the COVID crisis is emerging [5] looking at lifestyle behaviours associated with weight gain. It has been established that the pandemic reduced the perceived frequency of people engaging in behaviours associated with successful weight management and that people living with obesity and mental health problems may be at increased risk of pandemic‐related adverse health outcomes.

The National Institute for Health and Care Excellence (NICE) [6] published updated guidance for clinical assessment and management of obesity in 2014. This stated that bariatric surgery is the option of choice (instead of lifestyle interventions or drug treatment) for adults with a body mass index (BMI) of more than 50 kg/m^2^, when other interventions have not been effective. It recommended support for all those considering surgery, as well as, post‐operative follow‐up; including regular post‐operative assessment, management of co‐morbidities, psychological support before and after surgery, and information on or access to plastic surgery when appropriate. A review article [7] endorsed guidelines for the provision of psychological support pre‐ and post‐bariatric surgery using a stepped approach which takes account of the level of need for support. Research on the impact of commissioning weight loss surgery [8] noted the NHS England requirement that prior to bariatric surgery participants should have 12–24 months of specialist weight management support. It reported there is no supporting evidence for any such requirement and suggested that funding could be better spent on intensive lifestyle approaches rather than surgery. Reviews, specifically about lifestyle interventions, have identified ways in which these can be optimised. One [9] identified those that combined diet and exercise components achieved the greatest weight loss. A meta‐analysis [10] identified that behavioural treatment strategies improve adherence to a lifestyle intervention with significant positive effects found for session attendance and physical activity.

The experiences of patients before and after bariatric surgery were covered in a study [11] where patients were interviewed about their experiences of obesity before bariatric surgery to identify the nature and extent of the burden of obesity, including stigmatisation, shame and poor health. Participants expected surgery to result in ‘normality’: significant physical and psychological improvement. A systematic review of qualitative studies of patient experiences of bariatric surgery [12] concluded that the effect of bariatric surgery on psychological outcomes is complex, emphasising the need for health professionals to help patients manage the changes and challenges resulting from surgery by providing ongoing dietary and psychological support. A qualitative synthesis of current literature on patient experiences of healthcare professional‐led follow‐up from 12 months after bariatric surgery [13] pointed to a recurring theme for more and extended follow‐up, particularly psychological support. It identified access to plastic surgery for excess skin as a significant problem for a number of patients. Another study [14] found that patients generally described positive improvements in their physical, psychological and social state after weight loss surgery. However, patients also reported a number of unanticipated outcomes, such as adapting to a smaller body and changes in relationships. A study on the self‐evaluation of women following surgery [15] who found overall positive views but some negative issues with body image distortion and dissatisfaction. Looking at the support needs and experiences of patients after surgery [16]; a study identified peer, dietetic and psychological support as important factors influencing outcomes. In common with other studies, the authors found that psychological support was one of the most significant, but commonly overlooked, components of care. The key stage for professional involvement is in the first year after surgery. However, research [17] looking at the impact of giving patients access to three sessions with a health psychologist, compared to a control group who had treatment as usual, found no significant difference in BMI between those who had surgery and the psychological support and the control group who did not have this support after a year. They concluded that support should be targeted at patients who start to demonstrate weight gain at a later stage. This evidence contradicts other findings [18] which indicated that patients who attended six or more counselling sessions had better physical well‐being than those who attended less than six sessions.

**What is known about this topic?**

- Obesity is an increasing problem in the UK
- Lifestyle support can lead to significant weight loss
- Lifestyle support followed by bariatric surgery can also lead to significant weight loss

**What this study adds**

- Most participants had maintained their weight loss
- The experience had significantly improved the self‐esteem of some participants
- Weight loss came at a cost to some participants with problems over loose skin and restrictions on food intake

The concept of identity and changed identity through weight loss is raised in a number of articles. One describes identity [19] as a set of meanings which define the individual. People have a series of identities according to the situation they are in. For example, they can be a worker a spouse and a parent at the same time. A change in weight can lead to a change in health identity. Another study [20] looked at and suggested that more could be done to address the health outcomes of obesity rather than framing weight loss as the panacea for weight issues.

A study [21] argues that by reconfiguring their anatomy, patients aspire to adjust misalignments in the constitution of the embodied self that renders them incapable of moderate consumption. This issue was also discussed by another writer [22] who describes the experiences of patients after the ‘honeymoon’ period where the ‘work begins’ of maintaining their weight loss and a changed relationship with food starts. The author identifies components of dietary management, including changes in relationships with food and forming new habits. An article commenting on the outcomes of obesity surgery [23], in terms of weight loss, improved health and cost savings, says this does not describe the complexity of patient experiences and argues that the management of weight is always about more than health and simply having surgery to reduce weight. A study [24] examined the need to learn hunger and fullness after gastric bypass surgery. This followed an initial stage when participants were able to eat very little, to one where their eating patterns changed post‐surgery. Some aspects of eating needed to be adapted to accommodate their changed bodies. This included effects on their social life where they were no longer able to eat with family and friends in the ways that they had done pre‐surgery.

Overall, research on bariatric surgery and its long‐term impact are more plentiful than a lifestyle change approach. There is a complex interaction of factors influencing incidence, behaviours and engagement with support, lifestyle change, treatment and long terms sustained change and recovery. The results for the Tier 3 service provided by the team in this study mirror research findings [25] in that it works with two cohorts of patients—those who go through a lifestyle approach only and those who then go onto to have bariatric surgery. This study was conducted to look at the impact of a community‐based specialist weight management service for people with severe and complex obesity, to understand the long‐term effects of their involvement with the service and also to identify differences between the experiences of those who went on to have weight loss surgery from those who only had lifestyle support from the team.

## METHODS

### Study design and setting

This was an exploratory qualitative study using narrative interviews to understand the long‐term impact of contact with a Tier 3 weight management service.

The NHS in England provides services in a variety of settings varying from specialist in‐patient services in hospitals, often at a regional level, to services provided in people's homes on a one‐to‐one basis. The general practitioner service is provided in surgeries in local communities, such as parts of towns and villages, which run as stand‐alone businesses though these are largely funded by the NHS. Care, but not prescriptions, is free at the point of delivery and paid for from general taxatio**n**. Services for patients with weight management issues are provided at four levels.

Tier 1: Universal interventions (prevention and reinforcement of healthy eating and physical activity messages), which include public health and national campaigns, providing brief advice. This level of service is commissioned by local government.
Tier 2: Lifestyle weight management services, these are delivered by local community weight management services in non‐NHS settings (such as community or church halls or leisure centres). They provide a nutrition manual, lifestyle and behaviour change, education and support, in a group setting. People can usually self‐refer as well as be referred by a health or social care worker. Locally the service is provided by Derbyshire Community Healthcare NHS Foundation Trust and is open to patients with a BMI of over 25 or 23 from black or Asian ethnicity. The service is available on a time‐limited basis (usually 12 weeks) and is commissioned by the local authority.
Tier 3: Specialist Weight Management Services provide a clinician‐led NHS multidisciplinary team (MDT) approach, potentially including a physician (consultant or GP with a specialist interest), specialist nurse, specialist dietitian, psychologist, and physiotherapist. These services are commissioned and provided by the NHS and may be either community‐based or acute‐hospital‐based depending on local commissioning arrangements. Tier 3 services follow a clinical referral pathway with referrals coming from health practitioners. This service is usually provided on an individual basis for patients. It is normally time limited but over a longer term than Tier 2 services. At the time of this research, the service studied offered support for up to 2 years.
Tier 4: Surgical Bariatric Surgery, supported by a Clinician‐led MDT providing pre‐ and post‐operative care. The service is provided in a hospital and is commissioned by the NHS but elements of the care may be provided by the private sector.

The service is provided in a number of locations in community hospitals and clinics across the county. At the time the research took place some home visits were also being made. The team offers a comprehensive individual assessment and follow‐up including referral to surgery. It provides intensive individualised assessment and intervention for those with severe and complex obesity. Patients must have tried other weight‐reduction services and support at lower tiers of intervention and meet the criteria of a BMI of over 50, or a BMI of 35–49, with other health problems.  Referral is via a GP, practice nurse or any consultant seen at a hospital.

At the time participants were in contact with the service, access to medical expertise was through external referral. A holistic assessment is offered to begin the process of understanding each individual's own particular situation from a bio‐psychosocial perspective, and to inform the design of a lifestyle intervention programme to achieve sustainable weight loss. A weight management adviser (WMA) works intensively with the patient to support and implement lifestyle behaviour change. All patients have access to specialist dietetic support and a psychologist. The team receives clinical leadership from a clinical psychologist and the WMA's are supervised by psychological practitioners. There is a core psychology and behavioural insights approach to all lifestyle plans and interventions provide evidence‐based approaches to support sustained behavioural change with those engaging with the programme. At the time of the research participants were involved with the service there was also access to a physical activity specialist or physiotherapy. Support is provided for up to 1 year according to need. Patients eligible for and wishing to pursue bariatric surgery have to successfully complete a specified time with the service, depending on their BMI and health profile. Local commissioning requirements over the years since the service started have varied across time from between eight weeks to 9 months minimum pre‐surgery intervention. This timescale allows patients to engage with the same aspects of assessment and personalised lifestyle programme in order to provide them with detailed information about bariatric surgery and to introduce and begin to embed lifestyle behaviour change prior to the surgical intervention.

### Study participants

We identified records of all patients in touch with the weight management services from May 2012 onwards. From this group, those who had last had contact with the service more than 2 years ago were selected. A questionnaire was sent out in February 2018 to 448 patients. The questionnaire asked patients to indicate if they were interested in participating in a second interview stage to discuss their experiences in more detail. Questionnaires were received back from 102 patients, and of these, 37 patients expressed an initial interest in being interviewed. Those who expressed an interest in this stage were sent a letter outlining the purpose of the interview and a consent form. Twenty‐one consented to be interviewed. Two potential interviewees were unable to take part due to sickness and family commitments.

### Study tool

The interview guide was drawn up by the project team, but it was not piloted prior to use. It followed a narrative approach [26, 27, 28]. This approach allows questions to be asked with prompts to add detail to the topics. Some additional issues, such as the impact of loose skin, were added in light of the discussions that took place. The before and after service use data analysis of the research is reported separately [29].

The interview questions are shown in Table 1.

**TABLE 1:** Outline questions for patients who availed the National Health Service weight management services in the United Kingdom, interviewed in July–August 2018

Question	Stage asked
Lifestyle support without surgery	All interviews
Lifestyle support from the service followed by bariatric surgery	Bariatric surgery patients only
Effectiveness of surgery	Bariatric surgery patients only
Ways in which participants are now managing their weight	All interviews
Sources of support after leaving the service	All interviews
Key life events	All interviews
Facebook group for bariatric surgery patients	Added to schedule if issue came up in interviews
Loose skin	Added to schedule after if came up in interviews
Shame and embarrassment	All interviews
The meaning of food	All interviews
Changes in portion size	All interviews
Eating out	All interviews
Changes to levels of exercise	All interviews
Changes to mobility	Analysis of data for article

### Data collection

A group was established to run the project and met regularly. The team included the head of the service. The lead author was the interviewer and data analyst. He was formerly Research Manager for the NHS Trust where the study took place. He retired before the study started and worked on the project on an honorary basis as a Research Fellow. He had previously advised the service on research issues. He and the team were interested to understand the longer‐term impact of patient contact. He has master's degree level qualifications in research methods and public health. Feedback on the outputs of the study has been given to other members of the research team.

The interviews took place between June and August 2018. All the interviews took place in their homes. In some cases, family members were at home while the interviews took place but they did not take an active role in any of the discussions which were paused when they came into the room. The interviews were recorded, with participants’ agreement and transcribed. Participants were given a copy of their transcript to check before it was analysed. At this stage, one of the participants asked for significant changes to be made to the transcript before the analysis took place. No repeat interviews took place. The interviews took up to an hour.

### Data analysis

The interviewer was solely responsible for the classification of thematic analysis. NVivo software was used to sort data for analysis. The data on each topic was then read to identify common themes and areas of agreement and disagreement, which have been spelt out in the results. A question on changes in levels of the exercise was analysed for this article to give an understanding of changes in mobility and to provide a summary of the critical issues. The data presented is described in greater detail in the project report, which has formed the basis of some of the text used in this article.

### Ethics approval

Approval was obtained from South West ‐ Frenchay Research Ethics Committee in December 2017. No further changes were made to the study post‐approval. Those who took part were given a £20 supermarket gift voucher in recognition of their contributions. The use of the voucher was included in the ethics submission and was mentioned in the letter inviting participants to participate. It is not known if this had any influence on their decision to take part.

## RESULTS

### Profile of participants

There were 19 patients interviewed for this study. Seventeen were female; two were male: the disparity reflects the individuals willing to be interviewed. There is a gender bias in the referrals the service receives; 71% are female, and 29% are male. The average length of time participants had contact with the service was 81.9 days, compared to 85.7 days for all the patients in the study. The average score on the Index of Multiple Deprivation, which is a recognised UK measure of deprivation of study participants was 5.2, compared with 4.7 for all the patients in the study. Hence participants had on average spent less time with the service and lived in slightly less deprived areas than all patients contacted. There were no men who had undergone bariatric surgery. The details of the characteristics of patients interviewed for this study are shown in Table 2.

**TABLE 2:** Characteristics of patients who used a National Health Service weight management service in the United Kingdom, interviewed in July–August 2018

Participant serial no	Gender	Age group (years)	Living status	Employment status	Days with service	Lifestyle support OR Lifestyle support followed by surgery
1	Female	26–35	Divorced, living with partner	Further Education sector	65	Surgery
2	Female	26–35	Married with children	Retail supervisory	83	Surgery
3	Female	46–55	Married, adult children	National Health Service nursing/midwifery	113	Surgery
4	Female	56–65	Married	Recently made redundant from private company	99	Lifestyle
5	Female	26–35	Married children	National Health Service nursing/midwifery	82	Surgery
6	Female	46–55	Divorced children	Care sector unemployed	96	Surgery
7	Female	36–45	Divorced children	Care sector	84	Surgery
8	Male	46–55	Single	Disabled unable to work	93	Lifestyle
9	Female	26–35	Single parent children	Student	63	Proposed surgery did not take up
10	Female	56–65	Married,	Care sector	98	Lifestyle
11	Female	36–45	Married children	Care sector	114	Surgery
12	Female	26–35	Married children	Employed on maternity leave	91	Surgery
13	Female	46–55	Widowed adult children	Disabled unable to work	82	Surgery
14	Female	36–45	Married children	Disabled unable to work	62	Surgery, unable to take it up
15	Male	66–75	Married no children	Retired	91	Lifestyle
16	Female	46–55	Married adult children	Disabled unable to work	85	Lifestyle
17	Female	46–55	Divorced children	Disabled unable to work	65	Proposed surgery did not take up
18	Female	46–55	Married children	Self employed	43	Lifestyle
19	Female	26–35	Single parent children	Disabled unable to work	98	Surgery

### Lifestyle support without surgery

The support from the team gave one participant a better understanding of things like portion size and keeping a food diary [9]. However, keeping going was not always easy.

“Once you've learnt what you should do, you don't forget it…..You know I've took all that on board. But I'm just struggling at the minute maintaining anything because I've just got that much going on….” [Participant 16, Female]

There was a wish that support could be more open‐ended [15, 18]. Some problems were identified for one, support had not worked; they had put on weight again and become disheartened [10]. Another said they lacked the motivation to change their diet [4].

“….. it's regarded as a medical treatment, you don't (say to} anybody who's had cancer, ‘You've had 2 years, that's your lot.’ I think that definitely there should be more involvement with dietitians.” [Participant 15, Female]

One participant said that the support they had received worked if they followed the advice [8]. Another went to classes on a regular basis but did not gain much from the information sharing that took place in them [4].

### Lifestyle support from the service followed by bariatric surgery

All had some follow‐ups after surgery, but the amount and type of support varied. None was provided by the service. The support was through NHS hospitals rather than the private hospitals where the surgery took place under contract to NHS. Support from a dietitian was frequently mentioned [3, 6, 7, 11, 12, 19]. Other support included weighing [1, 3, 13] seeing the surgeon [11, 19]; physical health such as blood [1, 3]. One participant was still on the books of the service [2]. None had psychological support to discuss issues about coming to terms with a very different body shape and size.

One of the participants had helped to set up a Facebook blog for people who had had surgery [7]. This group had 3000 members, of whom about 20 were men. Another participant [19] found this a source of support.

“I came across this group and I thought,…..it's people who are going through the same thing’…. ‘Thank God I'm not on me own’, …So, it's nice to look on them posts and think, ‘Yeah they've done it, you know, I can get back on track”. [Participant 19, Female]

The issue of excess skin was a significant problem [1, 3, 6, 7, 11, 12, 13].

“The doctor said: don't you lose any more weight because the 15 pounds that you're carrying around that you don't like isn't fat it's skin. And I pack it all away in Lycra so it doesn't wobble”. [Participant 19, Female]

One participant [3] had surgery to remove it and others had considered it.

### Effectiveness of surgery

The surgery was successful for all but one participant [5, 12]. One had come off insulin onto oral medication and a diet to manage her diabetes [3]. However, there were problems, one issue was regaining weight [1, 19]. A number of participants reported problems post‐operation that significantly restricted their lifestyles.

“It worked well so it kept the weight off. The downside….. is that I had a stricture ….. and I've had a lot of stomach problems and (with) my bowels”. [Participant 11, Female]

“I have had a problem with my tummy. So, I've had my gallbladder out which was a, probably a reaction, ‘cos when you lose a lot of weight you create a lot of gallstones … they said some of the connective tissue that supports my bladder …, had a hole in it. So … subsequent of the operation it must have just come loose. … I've had a bit of pain issue occasionally”. [5]

Two participants said they had no restrictions on what they could eat.

*“Honestly, it's made …. absolutely no difference, to my eating… ”* [12]

### Other aspects of their experiences

The rest of the article looks at the experiences of participants who had lifestyle support and those who additionally had bariatric surgery. Where there are specific differences between the two groups, these are highlighted in the text.

### Ways in which all participants are now managing their weight

All but two of the participants were still doing something to manage their weight

“I have just done what I wanted to do,… we have steamed vegetables… we eat fish and chicken, we very rarely …. eat red meat, but I still have a sweet tooth. So, while our meals are exceedingly healthy most of the time, erm it's what … either away from home or after my… meal…I've put eight kilos on since the last time they weighed me, which is a considerable amount. …. but stress is always a factor, worry …..life doesn't get easier it seems…” [4]

“I tried to go back to Slimming World… there wasn't enough people attending… (So)I have just joined a gym … I'm hoping ….that'll ….prompt me to get going again”. [18]

Most of the participants said they tried to eat healthily every day [1, 2, 5, 6, 8, 9, 10, 11, 13, 16, 19], for example, one participant described what she ate in great detail and then said

*“I eat all the right good things and … I get plenty of exercise….; it manages my weight.”* [13]

A number found support through slimming groups [5, 7, 9, 10, 11, 12, 14, 18], but some had had been unable to keep this up [5, 14, 18]. Other approaches adopted were gym [1, 9, 12, 19]; support through other group/relative [2, 17]; walking [13, 15] and a diet sheet [1, 13]. Health problems prevented two participants from continuing activities [14, 19].

### Other aspects of life after contact with the service

Participants were asked about some key life events after leaving the service to understand if these impacted their continuing weight management. A number of participants had undertaken further education since their time with the service. One of them had done training related to weight management issues for work:

*“I've done…courses …regarding nutrition and healthy eating…..I was able …..to turn around and give it to my learners.”* [6]

One participant had taken on a role where she used her experience of weight management issues in her job advising other people about weight control:

“I have done a few bits about …educating women when they're pregnant …to maintain their weight or keep their weight down… so it has…enabled me to find out more (about) how I can help women that are overweight in pregnancy”. [3]

Another said she would like the opportunity to use her experiences in employment. She had already proof read some leaflets for organisations:

“I've …thought about learning about being a dietitian because I've got such a large knowledge of diets and food and what people go through….but nowhere locally do courses… I'd love to help people who've been through what I've been through…” [11]

Involvement with the service had not directly led to changes in employment. However, a number of participants, all from the surgery group [1, 2, 6, 7], said that because of their weight loss they had become more confident.

Participants were asked whether they took part in more exercise since they left the service. There was about an even split between those who said they were doing more [1‐3, 5, 7, 8, 11, 12, 13, 16]. Most of these participants had had surgery. Others said they were doing less [4, 10, 15, 17, 18, 19]. Most of these participants had been through the lifestyle approach. One participant [6] had been told not to do any exercise.

The related issue of changes in mobility came up in most interviews. Some of those said they were not able to be more mobile this was because they were constrained by health issues [6, 14]. For those who were more mobile the most dramatic illustration was one of the surgery participants

“After I lost the weight, the fact that I could get on a plane with the [Seat] arms down and a belt on and not worry about it, was probably the biggest thing that happened to me.” [1]

A number of participants reported fewer issues with shame and embarrassment, particularly those who had lost and maintained a significant weight loss.

“I walk with my head held high but someone said something about my arms the other day. It really upset me because I'm not heard anything (like that) for such a long time” [1]

For those who had not managed to lose weight or had regained it, problems still remained:

*“I caught sight of myself today. I look like a weeble on legs, and I didn't like that. There is some embarrassment and shame in that.”* [4]

Other views expressed included: issues with fitting clothes [4, 8, 10, 15]; feeling better since weight loss [3, 12, 15]; feeling more confident [1, 9, 12]; embarrassment increased due to weight gain [4, 10]; and continuing issues with other people's comments [1, 7].

### The meaning of food

Contact with the service made participants think about their relationship with food, in some cases in quite a profound way, although this had not led to changes in behaviour in every case. A number of participants felt that food now had a different meaning to them [5, 6, 7, 9, 11, 19]. All but one of these [9] were in the surgery group.

“Yes, I think I've taught myself not to see food in terms of good and bad……I love fruit and salad and all the best bits I eat it but if I'm craving a piece of chocolate I'll eat it … I've always known what I should be doing, just never done it”. [13]

Some participants said they had made changes in portion size [3, 16, 18, 19] and others said they had an increased awareness of calories in food [1, 4, 16, 17].

Participants also described quite a wide range of changes they had made in the type of food they ate. An increase in the amount of fruit and vegetables was the issue that recurred most frequently [4, 5, 6, 8, 9, 14, 15]. Changes in eating for pleasure or treats were discussed. Some said they still had something they fancied [2, 5, 8, 9, 16], and others specifically mentioned cake or biscuits [6, 7, 13, 17]. However, one participant no longer had treats.

*“I'd eat anything at any time of the day but now I don't really eat for pleasure anymore”*. [3]

Eating with others was an area where a number of participants had to make significant changes. Part of the issue for those who had surgery was that they no longer could eat as much as they used to and some foods became difficult to eat. For this group, another factor was whether participants had told the people they were going out with that they had undergone surgery [1, 7, 13, 19]:

“I didn't tell a lot of people that I was having the surgery… my family and friends knew,(about it) but they found it very difficult because… I can't go out for a meal and then go drinking” [1]

A number of participants said they rarely ate out [4, 8, 9, 17], and others they ate out less often [11, 12, 13]. Finally, two participants, both from the surgery group, spoke about people they knew not recognising them [1, 6]:

“My ex… walked past me a few times in the supermarket and he did not recognise me after twenty odd years together…people cannot believe ….how different I look and how cheerful I am…” [6]

## DISCUSSION

Involvement with an NHS specialist service providing intensive obesity management support for patients with severe and complex obesity had long‐term benefits for the participants. Most but not all maintained the weight loss they had achieved. For a number, their self‐esteem improved considerably. However, for some, the weight loss came with significant problems. One was a significant restriction on the amount they could eat or drink; in some cases, they could no longer eat certain types of food or drink. Some also had problems with excess skin. The support by the service was thought to be good, but there were issues with the amount of time available. For those who went on to have bariatric surgery, there was a particular issue about the lack of access to psychological support post‐surgery.

Many of the issues raised in the literature review were reflected in the findings of this study. For example, problems with excess skin raised in other research [15] and changed body image arose. The interview findings reflect further research about the complexity of the effect of bariatric surgery on psychological outcomes [12] which emphasises the need for health professionals to help patients manage the changes and challenges resulting from bariatric surgery by providing ongoing dietary and psychological support. The service provided both diet and exercise components and was thus in line with the recommended findings of one study [9]. Providing advice on physical activity meant the service also followed one of the findings of an article [10] about this improving adherence to a lifestyle intervention.

Participants talked about the need to continue closely monitoring what they eat. Some indicated a greater sense of normality by reporting that they no longer stood out in a crowd due to their weight. In these respects, the research findings support the conclusion of an article [11] which found that patients had a strong desire for a sense of normality post‐surgery. The research also indicates that weight management remains a struggle for many participants 2 years after leaving a Tier 3 specialist service. This suggests that services need to consider ways in which they can cater for participants who continue to have problems, even if only on a very short‐term basis. In this respect, the research supports the NICE guidance [6] on follow‐up after bariatric surgery. It would be, therefore, appropriate for commissioners to establish with providers what sort of support is provided and to what extent they follow up with patients to encourage them to stay in touch. This research also supports findings about the importance of psychological support [16]. The lack of this support for those undergoing bariatric surgery was an issue that several participants mentioned.

Bariatric surgery enabled participants to lose a lot of weight very quickly and made it easier for them to maintain the weight loss than possibly was the case with the lifestyle approach alone. Significant dietary restrictions and excess skin often followed the weight loss. These issues led to a degree of ambivalence about the impact of the surgery. So, this study finds some familiar narratives around the issues of coming to terms with a different body and the changes in identity which resulted from that [19, 20, 21]. There was also a need to change eating habits and relationships with food [22, 23]. Some participants expressed a hopeful narrative, whilst those who had more difficulty in maintaining weight loss fitted the weight cycling or stagnation narratives [20]. The impression gained from participants is that some did not feel adequately supported to cope with these changes after surgery, which echoes the findings of one study [13]. Findings about the need to change eating patterns post‐surgery to accommodate their changed bodies [24] are also supported. This included effects on their social life where they were no longer able to eat with family and friends in the ways that they had before, and the sense of loss that this caused.

### Weaknesses

The response rate to the initial questionnaire where they were invited to take part in the interviews was low, so those coming forward for interviews may not have been typical of the patients' experiences as a whole. The analysis of the interview data was carried out by the interviewer. It was not cross‐checked with other members of the research team due to the very limited resources available to undertake this project. The other members of the research team were involved in the framing of the research questions and also commented on the report and articles which came out of it.

## CONCLUSIONS

The interviews indicate a generally positive experience of the community Tier 3 Specialist Weight Management Service, qualified by a wish by several participants to have a longer or more open‐ended period of contact. Although participants who went on to have bariatric surgery reported a significant reduction in the number, it was at a high cost to some in terms of the amount and type of food they could eat. This impacted their social life. The problem of loose skin caused distress to several participants. So bariatric surgery outcomes can have a mixed impact on individuals with challenges that are difficult to adjust to and accommodate. Preparatory time spent with surgery patients helping them to understand the potential impact on their eating patterns, would help them to anticipate this cost. Specific discussions on the issues of loose skin and how to reduce its impact would also seem helpful. Finally, the provision of some access to psychological support post‐surgery also appears to be important.

## AUTHOR CONTRIBUTIONS

Mick Bond Honorary Research Fellow, School of Health and Related Research (ScHARR) University of Sheffield Regent Court Regent Street Sheffield S1 4DA email mick.bond@sheffield.ac.uk (Lead Author and address for correspondence). Mick Bond: Conceptualization; Data curation; Formal analysis; Investigation; Methodology; Project administration; Validation; Writing – original draft. Annette Haywood: Funding acquisition; Resources; Supervision; Writing – original draft; Writing – review & editing. Eleanor Holding: Writing – review & editing. Elizabeth Goyder: Funding acquisition; Resources. Paul Bissell: Conceptualization; Funding acquisition; Supervision; Writing – review & editing. John Stephenson: Formal analysis; Methodology; Writing – review & editing. Rachel Holt: Conceptualization; Data curation; Methodology; Project administration; Resources; Supervision; Writing – review & editing.

## CONFLICT OF INTEREST

The South West ‐ Frenchay Research Ethics Committee approved this study on 14^th^ December 2017 IRAS reference 172962. There have been no amendments to the protocol. All participants gave informed consent by returning the project questionnaire. At this stage participants were asked to indicate their interest in taking part in the second interview stage. Those who expressed an interest were sent further details. They were asked to complete and return a consent form to confirm their interest in taking part in the interview. Before the interviews started the consent was confirmed verbally and participants were advised that they could stop the interview at any stage. All participants were given a copy of the transcript of the interview to check and amend before its use in the study. All participants who asked to have a summary of the final report have been given one. There are no conflicts of interest for any of the authors listed below.

## Data Availability

Data available on request from the authors.
